# Strychnine as a Tonic

**Published:** 1853-07

**Authors:** 


*Strychnine as a Tonic.* By Dr. Parrish.—In a paper on this subject, Dr. Parrish records several cases in which marked benefit seemingly resulted from the use of the agent. Dr. P. thus concludes: Considering the specific action of strychnine as being directed to the nervous system, and the nervous system as the great centre of power, executing through all its multiplied divisions and ramifications the controling force which is necessary to preserve the harmony of organic life, it may well be inquired whether this remedy, though an active poison in overdoses, may not be judiciously employed in a variety of cases where a permanent tonic impression is needed. We have known it to do good in prolapsus uteri, depending upon a relaxed condition of the uterine ligaments and vaginal muscles, and it has done good by expending upon those relaxed parts its specific contractile influence. It is well known that, when given in paraplegia, for example, its effect will first be seen in the paralyzed limb. Its peculiar office seems to be to lay hold of those muscles which cannot act themselves, and stimulate them to normal effort. This action is, of course, produced through the nervous centres. Why, then, in cases of leuchorrhœa depending upon general debility of the nervous system, may it not act in the same way, and by the same rule? Why, in cases of prolapsed uterus depending upon a relaxation of the tissues retaining it naturally *in situ*, may it not produce and sustain that kind of *tonic spasm* necessary to restore it to its position, and hold it there? How else can the fact be explained? for cases of prolapsus uteri from debility have been treated by strychnine in tonic doses, with a success which pessaries and supporters could not secure.—*N. J. Med. Reporter.*

